# Which inhaled corticosteroid and long-acting β-agonist combination is better in patients with moderate-to-severe asthma, a dry powder inhaler or a pressurized metered-dose inhaler?

**DOI:** 10.1080/10717544.2017.1378937

**Published:** 2017-09-20

**Authors:** Masato Muraki, Kyuya Gose, Soichiro Hanada, Hirochiyo Sawaguchi, Yuji Tohda

**Affiliations:** a Department of Respiratory Medicine and Allergology, Kindai University Nara Hospital, Ikoma, Japan;; b Department of Respiratory Medicine and Allergology, Kindai University Faculty of Medicine, Osakasayama, Japan

**Keywords:** Asthma, dry powder inhaler, inhaled corticosteroid and long-acting β-agonist, inhaler device, pressurized metered-dose inhaler

## Abstract

Two main types of devices are used to facilitate the administration of inhaled corticosteroid (ICS) and long-acting β-agonist (LABA) in combination, dry powder inhalers (DPIs) and pressurized metered-dose inhalers (pMDIs). There are few reports comparing the effects of the two devices, and it is unknown which should be recommended for asthma patients with given sets of characteristics. In the current study, the beneficial effects and side effects associated with DPIs and pMDIs were compared, and the question of which device should be recommended for asthma patients was investigated. A prospective, randomized, crossover, comparative study in adult outpatients with asthma was conducted using salmeterol/fluticasone propionate combination (SFC) 50 μg/250 μg, one inhalation of Adoair^®^ 250 Diskus^®^ twice daily or two inhalations of Adoair^®^ 125 Aerosol twice daily, for 8 weeks. Questionnaires, exhaled nitric oxide (FeNO) tests and pulmonary function tests were administered after the use of each device for 8 weeks, and the results derived from each device were compared. Sixty-eight subjects were included in the final analysis. There were no significant differences between quality-of-life scores, FeNO, spirometry test results and forced oscillation results. With regard to patient preferences, 57.4% preferred the Adoair^®^ Aerosol and 35.3% preferred the Adoair^®^ Diskus^®^, as determined via the comparative evaluation questionnaire. Although DPI prescription accounts for the predominant market share of combined ICS/LABA in Japan, patients preferred a pMDI device to a DPI device. Compared to DPIs, pMDIs may be the preferential choice for patients with asthma.

## Introduction

In the Global Initiative for Asthma (GINA) report (Global Initiative for Asthma, 2017), the preferred controller of choice is inhaled corticosteroid (ICS) and long-acting β-agonist (LABA) combination for treatment step 3 or above. The availability of different ICS/LABA medication combinations and administration devices varies from country to country, and such devices include dry powder inhalers (DPIs) and pressurized metered-dose inhalers (pMDIs). DPIs are considered easier to use (Virchow et al., 2008). These devices also have limitations however, such as dependency of drug particle size on flow rate and loss of the metered dose if the patient exhales through the device before inhaling (Virchow et al., 2008). A relatively recent increase in the number of different types of inhaler devices available has resulted in a confusing number of choices for clinicians who are responsible for selecting delivery devices for individual patients (Dolovich et al., 2005).

Five types of devices and four ICS/LABA combinations are currently available in Japan, and only salmeterol/fluticasone propionate combination (SFC) is available in both DPI and pMDI devices. However, the differences in the beneficial effects and side effects between these two types of medications and the differences in patients’ preferences have not been elucidated, and it is unknown which device should be recommended for asthma patients with given sets of characteristics.

In the current prospective, randomized, crossover study, SFC DPIs and SFC pMDIs administering the same components and doses were compared in adult outpatients with asthma. The beneficial effects, side effects and patient preferences associated with the two devices were analyzed and compared in conjunction with various patient characteristics. A primary aim of the study was to shed some light on which devices should be recommended for asthma patients with specific sets of characteristics.

## Methods

Seventy-two outpatients aged ≥20 years with moderate-to-severe asthma who had been attending the Department of Respiratory Medicine and Allergology at Kindai University Nara Hospital (Ikoma, Japan) and who required treatment with a medium-dose ICS/LABA combination were initially enrolled in the study. Informed consent was obtained from all subjects, and the exclusion criteria were as follows: inability to inhale unassisted; inability to perform spirometry tests; pregnancy; severe comorbidities affecting quality of life (QoL) such as malignancy, cardiac failure, renal failure or severe liver dysfunction; and current smoker status. Subjects were randomly assigned to a ‘DPI preceding’ group (i.e. DPI first, then pMDI) or a ‘pMDI preceding’ group (pMDI first, then DPI), and their background characteristics were investigated (Supplementary Figure 1). The SFC (Adoair^®^, GlaxoSmithKline, Tokyo, Japan) regimens were inhalation of 1 blister (50 µg/250 µg) twice daily for the DPI (Adoair^®^ Diskus^®^) and inhalation of 2 puffs (50 µg/250 µg) twice daily for the pMDI (Adoair^®^ 125 Aerosol). No subject used a spacer such as AeroChamber^®^ for pMDI prior to the enrollment and no spacers were used for pMDI in this study.

The following evaluation items were investigated after the use of each device for 8 weeks: the Asthma Control Test (ACT) Questionnaire (Schatz et al., 2009; Thomas et al., 2009; Global Initiative for Asthma, 2017); the Asthma Health Questionnaire (AHQ)-33-Japan (Arioka et al., 2005; Muraki et al., 2008; Ichinose et al., 2017; Tohda et al., 2017); the original questionnaire for handling, side effects, adherence and overall evaluation (Supplementary Table 1); physical findings; fractional exhaled nitric oxide (FeNO) using NIOX MINO^®^ (Chest M.I., Tokyo, Japan) (Harnan et al., 2015); spirometry using a Chestac-33 (Chest M.I., Tokyo, Japan); and respiratory system resistance and reactance as determined via the forced oscillation technique (FOT) using MostGraph-01^®^ (Chest M.I., Tokyo, Japan) (Abe et al., 2016; Shirai and Kurosawa, 2016). When exacerbation due to a common cold or a similar cause overlapped with the visit date, the evaluation appointment was postponed for up to 4 weeks. The examinations were performed from 9 a.m. to 11 a.m., and 2–4 hours after the patients took their normal morning medications (SFC and all other morning medications). Use of short-acting β-agonist (SABA) within 12 hours of the visit was prohibited. Changes in concomitant medications were also prohibited from 8 weeks before the examination and throughout the study period. On the final day of the study, a questionnaire survey (Supplementary Table 2) to compare the SFC-DPI and SFC-pMDI was also administered. The study protocol was approved by the Institutional Review Board at Kindai University Nara Hospital and was implemented in compliance with the Declaration of Helsinki.

### Statistical analysis

Data are presented as the mean ± standard deviation. Statistical differences were assessed via the paired *t*-test for comparison between the two groups with correspondence, Student’s *t*-test for comparison between independent groups and Pearson’s goodness-of-fit test for comparison between categorical variables represented by a contingency table. With regard to device selection, odds ratio and confidence interval were calculated via logistic regression. Statistical analyses were performed using JMP^®^ version 10.0.2 statistical software (SAS Institute Japan, Tokyo, Japan), and *p* < .05 was considered statistically significant.

## Results

Seventy-two subjects were initially enrolled, and four of these subsequently dropped out – two who did not go to hospital on the final day of DPI administration in the pMDI preceding group, and one who revoked their consent and one who discontinued due to discomfort in the throat while using the pMDI in the DPI preceding group (Supplementary Figure 1). In the remaining 68 subjects, there was no overlap between the survey date and exacerbation due to a common cold or a similar cause, and all of them were able to take SFC during each of the 8-week periods. However, in 4 of the 68 subjects, FeNO could not be measured due to a technical problem. The characteristics of the 68 subjects included in the final analysis are shown in Table 1. In the final analysis, the DPI preceding group included 35 subjects and the pMDI preceding group included 33. Thirty-eight percent of the subjects had a history of smoking, and 57% had combined allergic rhinitis. The average ACT score was 22.8, and asthma was mostly well controlled as indicated by scores of ≥20 in 59 subjects (86.8%) and ≤19 in 9 subjects (13.2%). The mean grip strength of the fingers between the thumb and the forefinger and middle finger using a pinch meter was 7.0 kg, and all subjects could press a pMDI in order to self-administer a puff. Peak inspiratory flow (PIF) using the In-Check^®^ (Matsuyoshi & Co., Ltd., Tokyo, Japan) with an adapter for Diskus^®^ resistance (A/A/D) was higher than 30 L/min as a minimum of optimum PIF (van der Palen, 2003; Kawamatawong et al., 2017), except in one subject (29 L/min). All subjects have been using medium-dose ICS, and there were more DPI users than pMDI users (*n* = 45 for DPI, *n* = 20 for pMDI and *n* = 3 for both devices).

**Table 1.** Patient characteristics.

*N*	68	Pulmonary function test		
	Diskus preceding; 35	IC (L)	2.26 ± 0.63	
	Aerosol preceding; 33	FVC (L)	3.18 ± 0.95	
Male:female	32:36	%FVC (%)	101.8 ± 16.1	
Age (years old)	62.1 ± 15.6	FEV1 (L)	2.24 ± 0.71	
Height (cm)	161.6 ± 9.3	%FEV1 (%)	88.6 ± 17.6	
Body weight (kg)	62.2 ± 12.6	Post-BD FEV1 (L)	2.36 ± 0.73	
BMI (kg/m2)	23.7 ± 3.7	%Post-BD FEV1 (%)	92.9 ± 16.7	
Smoking	Never; 42 (61.8%)	Previous medications		
	Ex-smoker; 26 (38.2%)	ICS/LABA	SFC (DPI)	41
Pediatric asthma	12 (17.6%)		SFC (pMDI)	20
Disease duration (y)	10.5 ± 10.9		FBC	2
Allergic comorbidities	Allergic rhinitis; 39 (57.4%)		CIC + Sal	3
	Hay fever; 32 (47.1%)	ICS	Mo	1
			FP	1
ACT	22.8 ± 2.7 (median; 24)	Concomitant	LTRA	32
			TEO	4
			Other	12
Pinch meter (kg)	7.0 ± 2.1	Device type	pMDI	20
PIF (adaptor-free, L/min)	216.9 ± 77.7		DPI	45
(A/A/D, L/min)	92.3 ± 26.2		Both	3

BMI: body mass index; ACT: Asthma Control Test; A/A/D: adapter for Diskus^®^ resistance; PIF: peak inspiratory flow; IC: inspiratory capacity; FVC: forced vital capacity; FEV1: forced expiratory volume in the first second; BD: bronchodilator; ICS: inhaled corticosteroid; LABA: long-acting β-agonist; SFC: salmeterol/fluticasone combination; DPI: dry powder inhaler; pMDI: pressurized metered-dose inhaler; FBC: formoterol/budesonide combination (DPI); CIC: ciclesonide (pMDI); Sal: salmeterol (DPI); Mo: mometasone (DPI); FP: fluticasone propionate (DPI); LTRA: leukotriene receptor antagonists; TEO: theophylline.

Vital signs, FeNO levels and QoL scores (ACT and AHQ-33-Japan) after using the DPI or the pMDI for 8 weeks each are shown in Table 2. There were no significant differences in vital signs, FeNO levels or QoL scores between the DPI and pMDI datasets. An original questionnaire was also administered at the end of administration with each inhalation device (Table 3). Items 1 and 2 relate to handling and the inhalation process, the contents of the corresponding questions differ, and they were not a validated aspect of the comparison. Therefore, the differences between these items were not analyzed. With regard to items 4 and 5 however, the DPI seemed easier (item 4, *p* < .1; item 5, *p* = .0005). In side effect items, DPI was worse with respect to hoarseness (item 6), but this difference was not statistically significant. There were no significant differences between the results pertaining to any of the other items. In pulmonary function tests, there were no significant differences in spirometry parameters or MostGraph-01^®^ parameters (Table 4). There were also no significant differences in any MostGraph-01^®^ parameters at expiratory, inspiratory and expiratory minus inspiratory phases (Supplementary Table 3).

**Table 2.** Vital signs, FeNO levels and QoL scores (ACT and AHQ-33-Japan) after using the DPI or the pMDI.

	Adoair^®^ Diskus^®^	Adoair^®^ Aerosol	*p-*Value
Systolic BP (mmHg)	129.6 ± 17.2	130.3 ± 16.7	.3407
Diastolic BP (mmHg)	76.8 ± 9.6	77.1 ± 9.6	.3655
Pulse rate (/min)	79.5 ± 11.1	78.1 ± 11.4	.1190
FeNO (ppb)	28.9 ± 19.6	29.9 ± 25.4	.3304
ACT	23.1 ± 2.5	22.9 ± 2.4	.2857
AHQ-33-Japan			
AS	4.41 ± 4.05	4.75 ± 4.18	.2134
FWS	3.10 ± 3.88	3.32 ± 4.09	.2811
Em	3.07 ± 4.42	3.69 ± 4.25	.0504
DA	0.88 ± 1.43	1.01 ± 1.42	.2139
SA	0.93 ± 1.44	1.22 ± 1.90	.1013
Ec	0.51 ± 0.80	0.56 ± 0.89	.2762
Total	12.91 ± 11.90	14.54 ± 12.85	.0822
Face scale	0.99 ± 0.84	1.07 ± 0.74	.1383

FeNO: fractional exhaled nitric oxide; QoL: quality of life; ACT: Asthma Control Test; AHQ: Asthma Health Questionnaire; DPI: dry powder inhaler; pMDI: pressurized metered-dose inhaler; BP: blood pressure; ACT: Asthma Control Test; AS: asthma symptoms; FWS: factors which worsened symptoms; Em: emotion; DA: daily activity; SA: social activity; Ec: economic.

**Table 3.** Scores on the original questionnaire after using the DPI or the pMDI.

	Adoair® Diskus®	Adoair® Aerosol	*p-*Value
1) Handling	1.38 ± 0.60	1.79 ± 1.04	N.A.
2) Inhalation technique	1.62 ± 0.79	1.62 ± 0.71	N.A.
3) Mouthwash	1.40 ± 0.74	1.35 ± 0.62	.2584
4) Inhale well?	1.75 ± 0.82	1.88 ± 0.76	.0957
5) Difficulty of handling	1.65 ± 0.71	1.99 ± 0.80	.0005
6) Hoarseness, dysphonia	2.26 ± 1.28	2.06 ± 1.08	.0661
7) Throat irritation	1.66 ± 0.84	1.75 ± 0.92	.2001
8) Discomfort in the throat	1.93 ± 1.03	1.94 ± 1.09	.4636
9) Cough immediately after inhalation	1.5 ± 0.84	1.5 ± 0.76	.5000
10) Aftertaste	1.78 ± 0.79	1.90 ± 0.94	.1566
11) Headache	1.24 ± 0.55	1.17 ± 0.49	.2091
12) Palpitation	1.24 ± 0.58	1.18 ± 0.52	.2418
13) Tremor	1.18 ± 0.42	1.18 ± 0.62	.5000
14) Adherence	1.76 ± 0.79	1.82 ± 0.86	.2877
15) Overall evaluation	2.18 ± 0.69	2.22 ± 0.73	.3445

DPI: dry powder inhaler; pMDI: pressurized metered-dose inhaler; N.A.: not assessed.

**Table 4.** Spirometry and MostGraph-01^®^ after using the DPI or the pMDI.

Spirometry	Adoair^®^ Diskus^®^	Adoair^®^ Aerosol	*p-*Value
IC (L)	2.30 ± 0.66	2.29 ± 0.63	.6168
FVC (L)	3.22 ± 0.98	3.23 ± 0.98	.3878
FEV1 (L)	2.29 ± 0.72	2.30 ± 0.74	.4116
PEF (L/s)	6.37 ± 1.75	6.36 ± 1.80	.5619
FEF50% (L/s)	2.32 ± 1.24	2.31 ± 1.25	.6174
FEF25% (L/s)	0.62 ± 0.46	0.62 ± 0.49	.4976
FEF50%/FEF25%	4.29 ± 1.22	4.33 ± 1.36	.4024
MostGraph-01^®^ (mean)			
R5	3.36 ± 1.43	3.35 ± 1.29	.5627
R20	2.82 ± 1.03	2.85 ± 0.98	.3195
R5-R20	0.54 ± 0.52	0.49 ± 0.46	.8491
X5	−0.79 ± 1.15	−0.78 ± 0.81	.4806
Fres	9.27 ± 4.29	9.50 ± 3.78	.2597
ALX	4.87 ± 11.70	4.34 ± 6.80	.7029

DPI: dry powder inhaler; pMDI: pressurized metered-dose inhaler; IC: inspiratory capacity; FVC: forced vital capacity; FEV1: forced expiratory volume in the first second; PEF: peak expiratory flow; FEF: forced expiratory flow; R5: resistance at 5 Hz; R20: resistance at 20 Hz; X5: reactance at 5 Hz; Fres: frequency of resonance; ALX: low-frequency reactance area.

**Figure 1.**
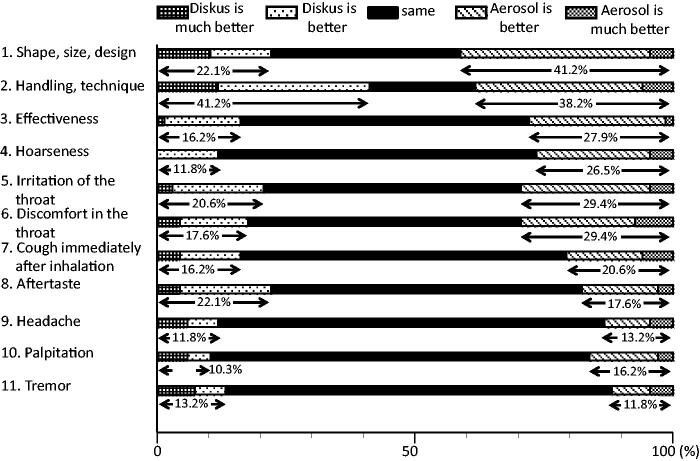
Questionnaire administered at the end of the study in which the subjects compared the DPI and the pMDI.

**Figure 2.**
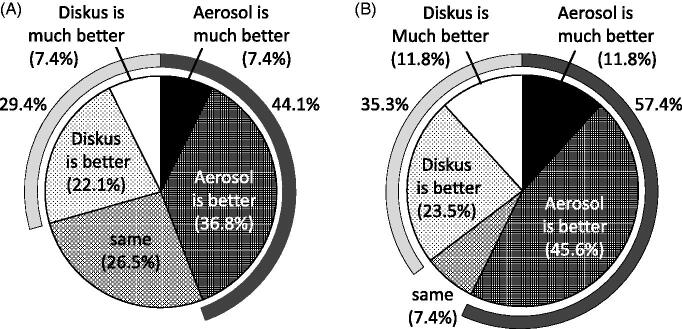
Questionnaire administered at the end of study, in which the subjects provided their overall evaluations and preferences for the DPI and the pMDI. (A) Overall evaluation. (B) The ‘Which would you like to use from now?’ item.

At the end of the study, a questionnaire comparing the DPI and the pMDI was administered (Figures 1 and 2). With respect to the shape, size and design of the devices, 41.2% of patients reported that the pMDI was better. In the items relating to side effects, the DPI was worse with regard to hoarseness, irritation of the throat and discomfort in the throat (DPI better, 11.8–20.6%; pMDI better, 26.5–29.4%). Other side effects were approximately equivalent (Figure 1). In overall evaluations, 44.1% of the subjects preferred the pMDI, and 29.4% preferred the DPI (Figure 2(A)). In addition, in response to an item asking patients which device they would like to use from now, 57.4% nominated the pMDI, and 35.3% nominated the DPI (Figure 2(B)).

In an effort to identify factors affecting device preference, sex, age, height, weight, body mass index (BMI), PIF, presence/absence of pediatric asthma, disease duration, smoking history, ACT score and spirometric parameters were compared in the 24 subjects who preferred the DPI and the 39 who preferred the pMDI. However, there were no significant differences in any of these factors (Supplementary Table 4). The odds ratios associated with preferring the pMDI to the DPI were calculated for sex (female), age (≥65 years), BMI (≥25), PIF using an adapter for Diskus^®^ resistance (<45 L/min), smoking history (presence), history of pediatric asthma (presence), disease duration (<10 years), comorbid allergic rhinitis (presence), ACT score (≥20) and spirometric parameters including inspiratory capacity <2 L, forced vital capacity <80%, forced expiratory volume in the first second (FEV1) <80% and post-bronchodilator FEV1 ≤ 80% (Supplementary Table 5). None of these odds ratios were statistically significant. The proportion of previous DPI users who nominated the pMDI device was 53.3% (24 subjects, 18 subjects for DPI better and 3 subjects for equivalent), and the proportion of previous pMDI users who nominated the pMDI device was 65% (13 subjects, 5 subjects for DPI better and 2 for equivalent), and this difference was not statistically significant (*p* = .2709).

## Discussion

Even though inhalation therapy is a cornerstone in asthma and chronic obstructive pulmonary disease management, little advice is available in the relevant guidelines relating to inhaler selection. A relatively recent increase in the number of different inhaler devices available has resulted in a confusing number of choices for clinicians who are responsible for selecting delivery devices for individual patients (Dolovich et al., 2005). Two main types of devices are available for the delivery of ICS and LABA combined, DPIs and pMDIs, and in Japan only SFC (Adoair^®^) is available in both types. In a previous study, Adoair^®^ DPI (Diskus^®^) and Adoair® pMDI (Aerosol) exhibited equivalence with regard to the administration of high-dose SFC (van Noord et al., 2001). However, in that report there was no consideration of which devices should be recommended for which kinds of patients, or patient preferences.

In the present study, there were no significant differences in physical examination parameters (blood pressure and pulse rate), FeNO levels, health-related QoL (ACT and AHQ-33-Japan), spirometric parameters or MostGraph-01^®^ parameters associated with the DPI and the pMDI after each treatment. In a previous retrospective study, it was reported that patients in a pMDI cohort were more likely to achieve asthma control and treatment success (defined as no exacerbation and no change in asthma therapy) than a DPI cohort (Price et al., 2011). In a different study however, FeNO levels were improved to similar degrees in a comparison of DPI- and MDI-administered mometasone furoate (Nolte et al., 2013).

Although several improvements in pMDIs such as changes in the propellant and actuation have resulted in improvements in lung deposition, many DPIs are easier to use (Virchow et al., 2008). As compared with the DPI, the pMDI requires greater effort to synchronize the puff and the inhalation, and it has been suggested that the inhalation error rate is high, particularly as compared to the low DPI misoperation rate (Molimard et al., 2003). In the present study, the DPI was also associated with better results with regard to handling, as determined via the original questionnaire. This may be due to the relatively simple operation of the DPI itself, but notably, the previous DPI users in the study were likely to have already become accustomed to using the DPI prior to the commencement of the study.

At the end of each treatment, hoarseness associated with the pMDI was weaker than that associated with the DPI, though this difference was not statistically significant. In the final comparative questionnaire, pharyngolaryngeal side effects including hoarseness were also more strongly associated with the DPI than the pMDI. The number of subjects who reported that the pMDI was more effective than the DPI was higher than the number of subjects who reported the reverse, despite the fact that there were no significant differences in FeNO levels or any of the pulmonary function parameters tested. In addition, in the overall evaluation more subjects reported that they preferred the pMDI. Although the reason why more subjects preferred the pMDI could not be elucidated, it was evidently not simply that a new device was preferred, because the ratio of previous DPI users who nominated the pMDI device was less than the ratio of previous pMDI users who nominated pMDI device.

Inhaler devices may influence patient compliance with long-term asthma medication regimens (Darbà et al., 2016). pMDI devices such as those used for ICS/LABA initial treatment are associated with longer treatment persistence and better treatment adherence in asthma, as well as lower exacerbation rates, reduced use of health resources and lower costs (Sicras et al., 2016). We studied pMDIs without a spacer in the present study. However, pMDIs may prove more effective – especially in elderly patients – if they are used with a spacer. Higher therapeutic efficacy has been reported with the use of a pMDI with a spacer for fluticasone inhalation than for DPI in post-elementary school-aged patients (Miyahara et al., 2008). Notably however, according to individual patients, the additional use of a spacer may hinder adherence. Therefore, it is necessary to consider the characteristics and needs of individual patients.

The characteristics of patients who preferred DPI and those who preferred pMDI were compared, but no significant differences were detected. The pMDI is still the most frequently prescribed device worldwide, but even after repeated tuition many patients fail to use it correctly (Virchow et al., 2008). Notably, the device market share in Japan is dominated by the DPI. The current Japanese ICS/LABA combination is predominantly accounted for by the DPI, and the numbers of prescriptions for outpatients are 14,015,551 (equating to 78.155 billion yen) for the DPI and 1,244,337 (equating to 6.494 billion yen) for the pMDI (AnswersNews, http://answers.ten-navi.com/pharmanews/7962). We cannot fully explain the reason DPI dominates the market share in Japan. However, in part it may be related to the lower malfunction rate and shorter instruction time necessary for a DPI. In addition, the preferences of medical professionals may be different than those of patients.

A limitation of the present study is that the device used for rapid relief with rescue SABA inhalers, DPI or pMDI, was not regulated in the present study, which may have influenced subject preference. In addition, the subjects in this study all had severe asthma requiring medium-dose ICS/LABA, so our findings may not be applicable to patients with different severities of asthma.

The current study investigated patients’ preferences with regard to SFC (Adoair^®^) DPIs and pMDIs. Despite the current Japanese market share distribution, patients with asthma tended to prefer the pMDI to the DPI. Therefore, prescribing the pMDI may be preferable, considering patients’ preferences. Further investigation of the selection of the DPI or the pMDI for individual patients is necessary in the future.

## Supplementary Material

IDRD_Muraki_et_al_Supplemental_Content.pptx

## References

[CIT0001] Abe Y, Shibata Y, Igarashi A, et al. (2016). Reference values of MostGraph measures for middle-aged and elderly Japanese individuals who participated in annual health checkups. Respir Investig 54:148–55. 10.1016/j.resinv.2015.12.004 27108009

[CIT0002] Arioka H, Kobayashi K, Kudo K, et al. (2005). Validation study of a disease-specific module, the Asthma Health Questionnaire (AHQ) using Japanese adult asthmatic patients. Allergol Int 54:473–82.

[CIT0003] Darbà J, Ramírez G, Sicras A, et al. (2016). Identification of factors involved in medication compliance: incorrect inhaler technique of asthma treatment leads to poor compliance. Patient Prefer Adherence 10:135–45. 26929605 10.2147/PPA.S95303 PMC4754100

[CIT0004] Dolovich MB, Ahrens RC, Hess DR, et al. (2005). Device selection and outcomes of aerosol therapy: evidence-based guidelines: American College of Chest Physicians/American College of Asthma, Allergy, and Immunology. Chest 127:335–71. 15654001 10.1378/chest.127.1.335

[CIT0005] Global Initiative for Asthma (GINA). 2017. GINA Report, Global Strategy for Asthma Management and Prevention. Available at: http://ginasthma.org.

[CIT0006] Harnan SE, Tappenden P, Essat M, et al. (2015). Measurement of exhaled nitric oxide concentration in asthma: a systematic review and economic evaluation of NIOX MINO, NIOX VERO and NObreath. Health Technol Assess 19:1–330. 10.3310/hta19820 PMC4781557 26484874

[CIT0007] Ichinose M, Sugiura H, Nagase H, et al. (2017). Japanese guidelines for adult asthma 2017. Allergol Int 66:163–89. 28196638 10.1016/j.alit.2016.12.005

[CIT0008] Kawamatawong T, Khiawwan S, Pornsuriyasak P. (2017). Peak inspiratory flow rate measurement by using In-Check DIAL for the different inhaler devices in elderly with obstructive airway diseases. J Asthma Allergy 10:17–21. 28260934 10.2147/JAA.S127580 PMC5328129

[CIT0009] Miyahara H, Korematsu S, Nagakura T, et al. (2008). Efficacy of fluticasone metered-dose inhaler and dry powder inhaler for pediatric asthma. Pediatr Int 50:103–8. 18279216 10.1111/j.1442-200X.2007.02523.x

[CIT0010] Molimard M, Raherison C, Lignot S, et al. (2003). Assessment of handling of inhaler devices in real life: an observational study in 3811 patients in primary care. J Aerosol Med 16:249–54. 14572322 10.1089/089426803769017613

[CIT0011] Muraki M, Ichihashi H, Haraguchi R, et al. (2008). Comparison of the Asthma Health Questionnaire-33-Japan and the short-form 36-item health survey for measuring quality of life in Japanese patients with asthma. Allergol Int 57:339–46. 18690008 10.2332/allergolint.O-07-516

[CIT0012] Nolte H, Pavord I, Backer V, et al. (2013). Dose-dependent anti-inflammatory effect of inhaled mometasone furoate/formoterol in subjects with asthma. Respir Med 107:656–64. 23490226 10.1016/j.rmed.2013.02.010

[CIT0013] Price D, Roche N, Christian Virchow J, et al. (2011). Device type and real-world effectiveness of asthma combination therapy: an observational study. Respir Med 105:1457–66. 21612903 10.1016/j.rmed.2011.04.010

[CIT0014] Schatz M, Kosinski M, Yarlas AS, et al. (2009). The minimally important difference of the Asthma Control Test. J Allergy Clin Immunol 124:719–23. 19767070 10.1016/j.jaci.2009.06.053

[CIT0015] Shirai T, Kurosawa H. (2016). Clinical application of the forced oscillation technique. Intern Med 55:559–66. 26984069 10.2169/internalmedicine.55.5876

[CIT0016] Sicras A, Ferrer V, Collar JM, et al. (2016). Persistence to treatment by type of inhaler device in patients with asthma and chronic obstructive pulmonary disease. Semergen 43:375–86. [Spanish] 27425817 10.1016/j.semerg.2016.05.008

[CIT0017] Thomas M, Kay S, Pike J, et al. (2009). The Asthma Control Test (ACT) as a predictor of GINA guideline-defined asthma control: analysis of a multinational cross-sectional survey. Prim Care Respir J 18:41–9. 19240948 10.4104/pcrj.2009.00010 PMC6619040

[CIT0018] Tohda Y, Iwanaga T, Sano H, et al. (2017). Improved quality of life in asthma patients under long-term therapy: assessed by AHQ-Japan. Int J Clin Pract 71:e12898. 10.1111/ijcp.12898 27933734

[CIT0019] van der Palen J. (2003). Peak inspiratory flow through Diskus and Turbuhaler, measured by means of a peak inspiratory flow meter (In-Check DIAL). Respir Med 97:285–9. 12645837 10.1053/rmed.2003.1289

[CIT0020] van Noord JA, Lill H, Carillo Diaz T, et al. (2001). Clinical equivalence of a salmeterol/fluticasone propionate combination product (50/500µg) delivered via a chlorofluorocarbon-free metered-dose inhaler with the Diskus™ in patients with moderate to severe asthma. Clin Drug Invest 21:243–55.

[CIT0021] Virchow JC, Crompton GK, Dal Negro R, et al. (2008). Importance of inhaler devices in the management of airway disease. Respir Med 102:10–19. 17923402 10.1016/j.rmed.2007.07.031

