# Supplementary material for: Which inhaled corticosteroid and long-acting β-agonist combination is better in patients with moderate-to-severe asthma, a dry powder inhaler or a pressurized metered-dose inhaler?
Source: Drug Deliv. 2017 Sep 20;24(1):1395–400. doi: 10.1080/10717544.2017.1378937 (PMC8241158; doi:10.1080/10717544.2017.1378937)
Supplement: IDRD_Muraki_et_al_Supplemental_Content.pptx [file IDRD_A_1378937_SM0059.pptx]

## Slide 1
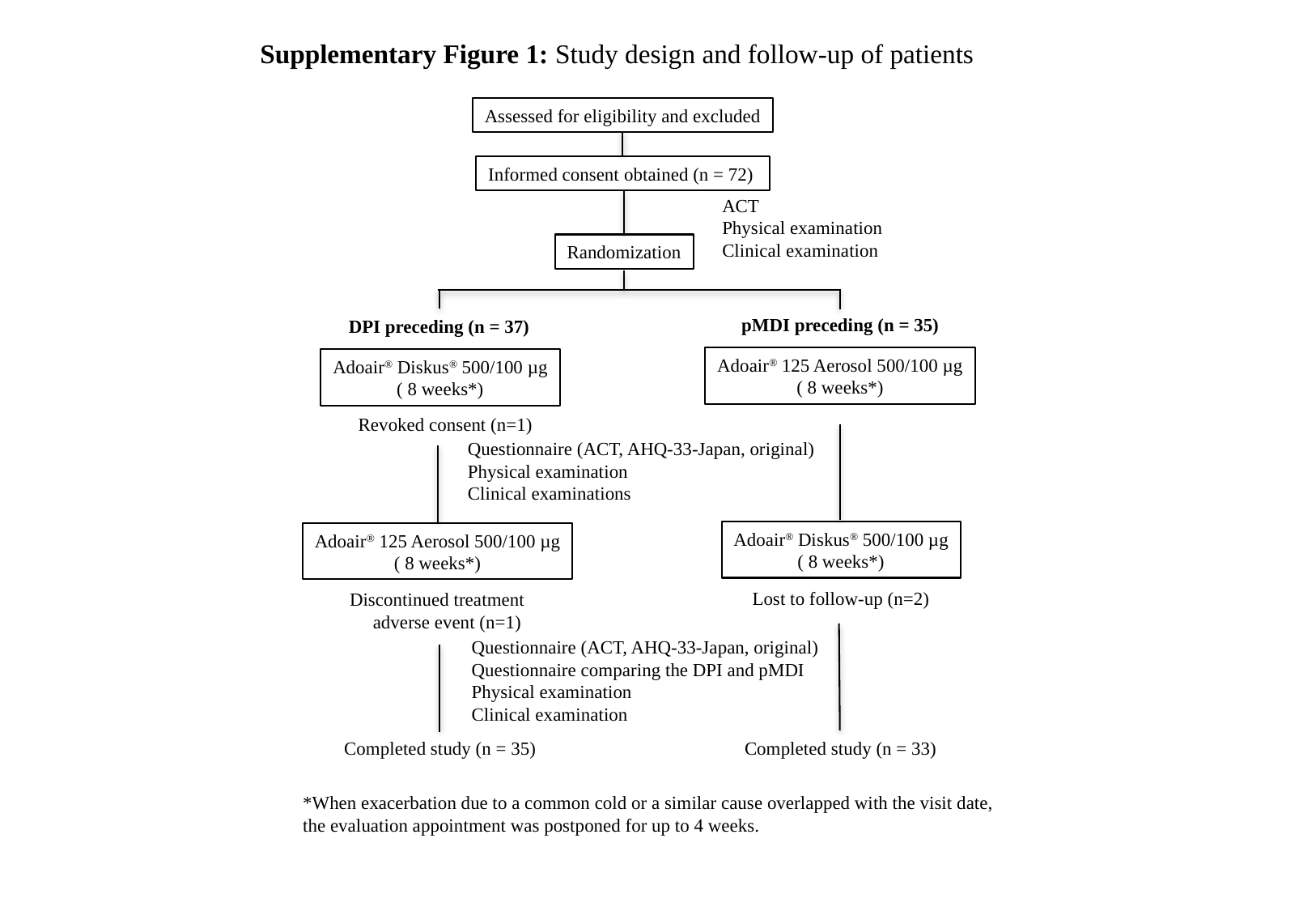

Supplementary Figure 1: Study design and follow-up of patients
Assessed for eligibility and excluded
Informed consent obtained (n = 72)
ACT
Physical examination
Clinical examination
Randomization
pMDI preceding (n = 35)
DPI preceding (n = 37)
Adoair® 125 Aerosol 500/100 µg
( 8 weeks*)
Adoair® Diskus® 500/100 µg
( 8 weeks*)
Revoked consent (n=1)
Questionnaire (ACT, AHQ-33-Japan, original)
Physical examination
Clinical examinations
Adoair® Diskus® 500/100 µg
( 8 weeks*)
Adoair® 125 Aerosol 500/100 µg
( 8 weeks*)
Lost to follow-up (n=2)
Discontinued treatment
 adverse event (n=1)
Questionnaire (ACT, AHQ-33-Japan, original)
Questionnaire comparing the DPI and pMDI
Physical examination
Clinical examination
Completed study (n = 35)
Completed study (n = 33)
*When exacerbation due to a common cold or a similar cause overlapped with the visit date, the evaluation appointment was postponed for up to 4 weeks.

## Slide 2
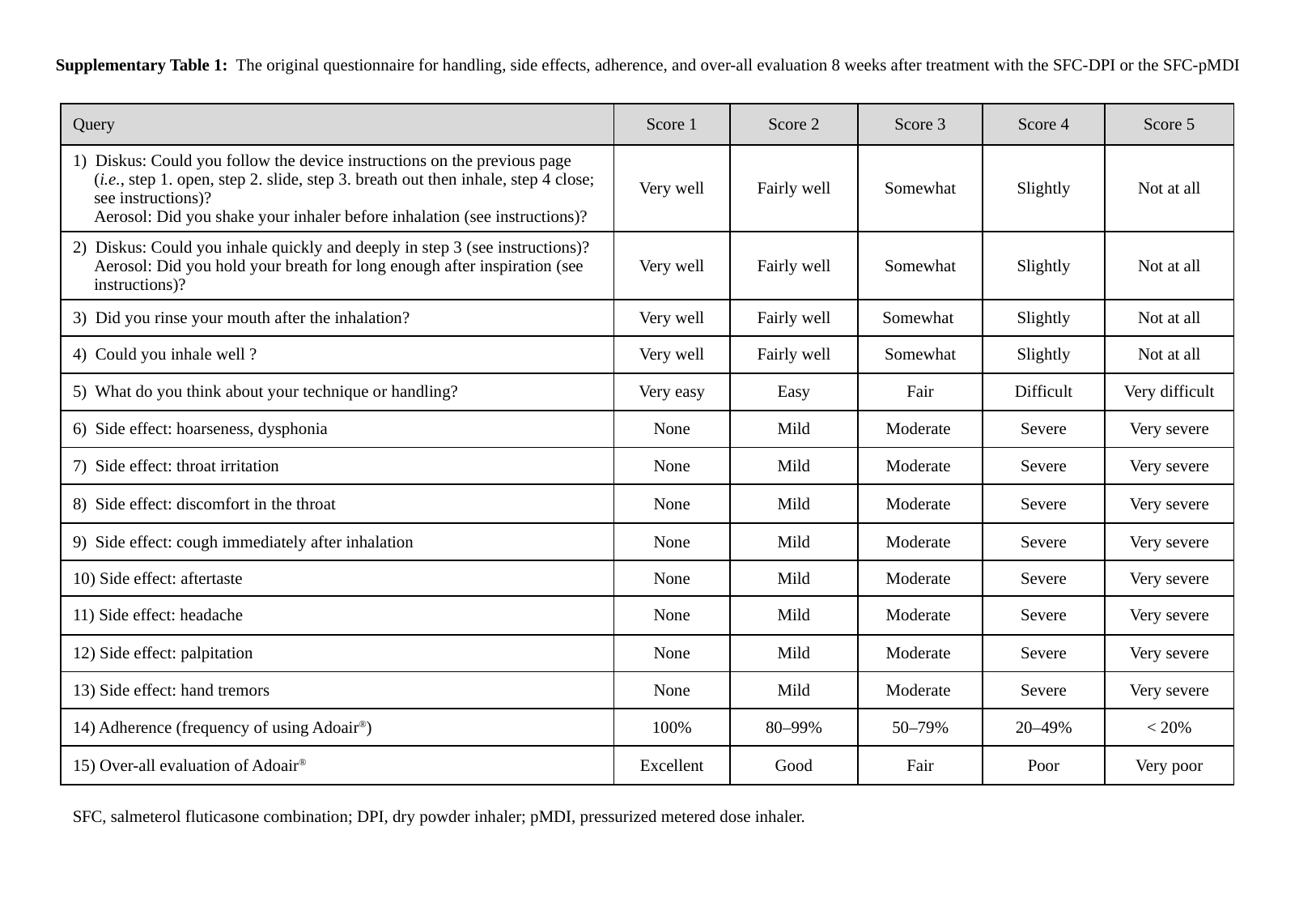

Supplementary Table 1: The original questionnaire for handling, side effects, adherence, and over-all evaluation 8 weeks after treatment with the SFC-DPI or the SFC-pMDI
| Query | Score 1 | Score 2 | Score 3 | Score 4 | Score 5 |
| --- | --- | --- | --- | --- | --- |
| 1) Diskus: Could you follow the device instructions on the previous page (i.e., step 1. open, step 2. slide, step 3. breath out then inhale, step 4 close; see instructions)? Aerosol: Did you shake your inhaler before inhalation (see instructions)? | Very well | Fairly well | Somewhat | Slightly | Not at all |
| 2) Diskus: Could you inhale quickly and deeply in step 3 (see instructions)? Aerosol: Did you hold your breath for long enough after inspiration (see instructions)? | Very well | Fairly well | Somewhat | Slightly | Not at all |
| 3) Did you rinse your mouth after the inhalation? | Very well | Fairly well | Somewhat | Slightly | Not at all |
| 4) Could you inhale well ? | Very well | Fairly well | Somewhat | Slightly | Not at all |
| 5) What do you think about your technique or handling? | Very easy | Easy | Fair | Difficult | Very difficult |
| 6) Side effect: hoarseness, dysphonia | None | Mild | Moderate | Severe | Very severe |
| 7) Side effect: throat irritation | None | Mild | Moderate | Severe | Very severe |
| 8) Side effect: discomfort in the throat | None | Mild | Moderate | Severe | Very severe |
| 9) Side effect: cough immediately after inhalation | None | Mild | Moderate | Severe | Very severe |
| 10) Side effect: aftertaste | None | Mild | Moderate | Severe | Very severe |
| 11) Side effect: headache | None | Mild | Moderate | Severe | Very severe |
| 12) Side effect: palpitation | None | Mild | Moderate | Severe | Very severe |
| 13) Side effect: hand tremors | None | Mild | Moderate | Severe | Very severe |
| 14) Adherence (frequency of using Adoair®) | 100% | 80–99% | 50–79% | 20–49% | < 20% |
| 15) Over-all evaluation of Adoair® | Excellent | Good | Fair | Poor | Very poor |
SFC, salmeterol fluticasone combination; DPI, dry powder inhaler; pMDI, pressurized metered dose inhaler.

## Slide 3
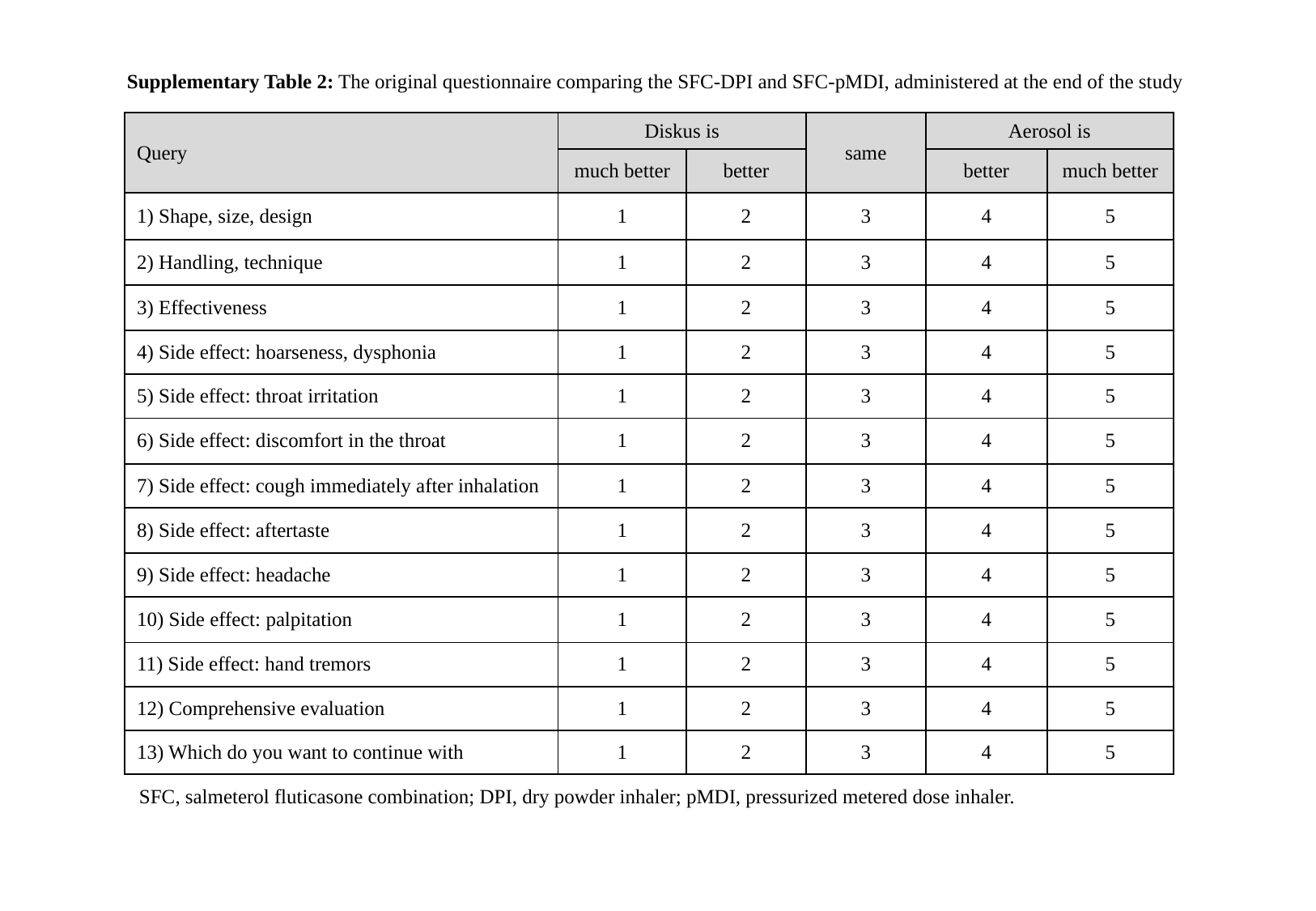

Supplementary Table 2: The original questionnaire comparing the SFC-DPI and SFC-pMDI, administered at the end of the study
| Query | Diskus is | | same | Aerosol is | |
| --- | --- | --- | --- | --- | --- |
| | much better | better | | better | much better |
| 1) Shape, size, design | 1 | 2 | 3 | 4 | 5 |
| 2) Handling, technique | 1 | 2 | 3 | 4 | 5 |
| 3) Effectiveness | 1 | 2 | 3 | 4 | 5 |
| 4) Side effect: hoarseness, dysphonia | 1 | 2 | 3 | 4 | 5 |
| 5) Side effect: throat irritation | 1 | 2 | 3 | 4 | 5 |
| 6) Side effect: discomfort in the throat | 1 | 2 | 3 | 4 | 5 |
| 7) Side effect: cough immediately after inhalation | 1 | 2 | 3 | 4 | 5 |
| 8) Side effect: aftertaste | 1 | 2 | 3 | 4 | 5 |
| 9) Side effect: headache | 1 | 2 | 3 | 4 | 5 |
| 10) Side effect: palpitation | 1 | 2 | 3 | 4 | 5 |
| 11) Side effect: hand tremors | 1 | 2 | 3 | 4 | 5 |
| 12) Comprehensive evaluation | 1 | 2 | 3 | 4 | 5 |
| 13) Which do you want to continue with | 1 | 2 | 3 | 4 | 5 |
SFC, salmeterol fluticasone combination; DPI, dry powder inhaler; pMDI, pressurized metered dose inhaler.

## Slide 4
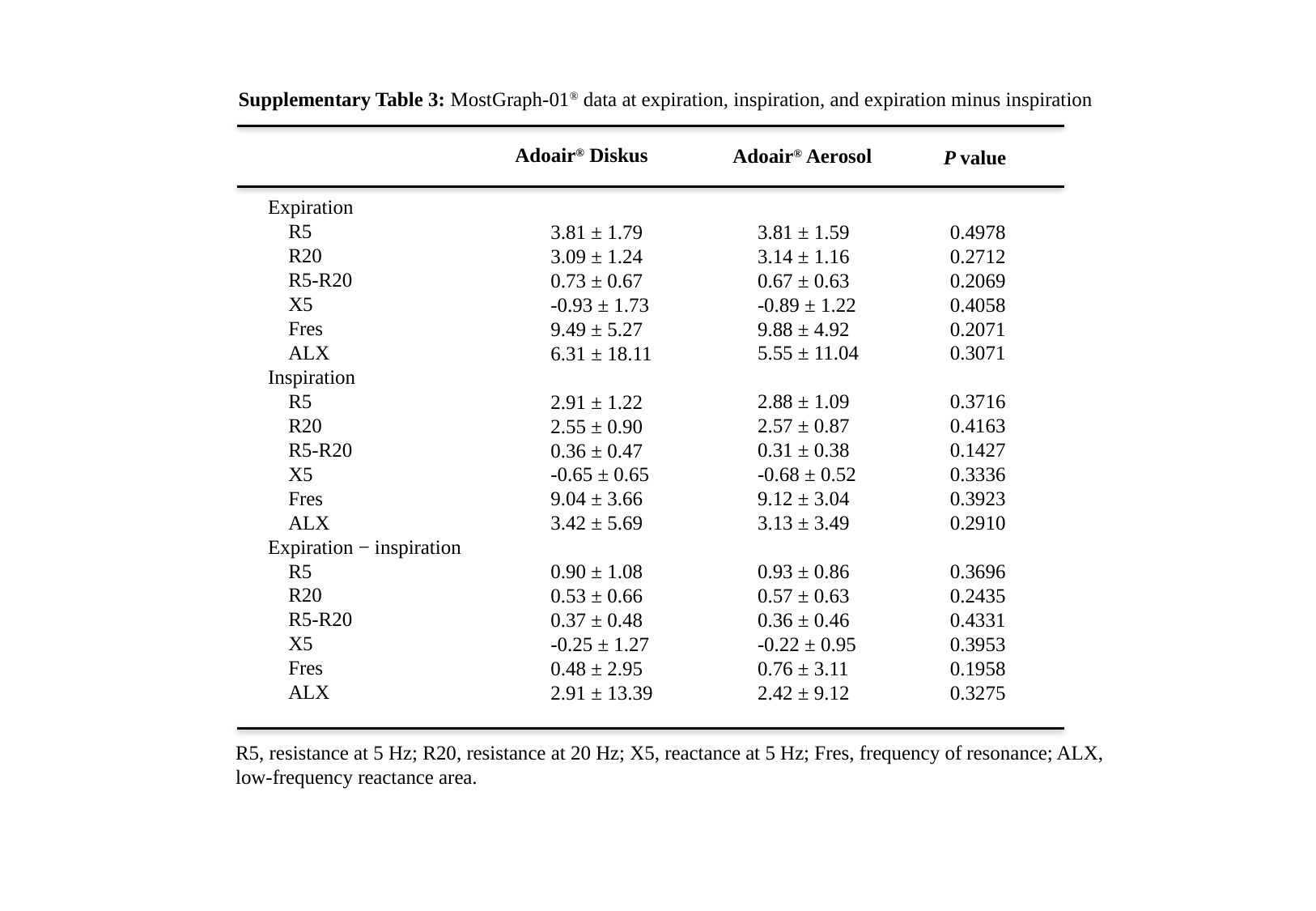

Supplementary Table 3: MostGraph-01® data at expiration, inspiration, and expiration minus inspiration
Adoair® Diskus
Adoair® Aerosol
P value
Expiration
 R5
 R20
 R5-R20
 X5
 Fres
 ALX
Inspiration
 R5
 R20
 R5-R20
 X5
 Fres
 ALX
Expiration − inspiration
 R5
 R20
 R5-R20
 X5
 Fres
 ALX
3.81 ± 1.59
3.14 ± 1.16
0.67 ± 0.63
-0.89 ± 1.22
9.88 ± 4.92
5.55 ± 11.04
2.88 ± 1.09
2.57 ± 0.87
0.31 ± 0.38
-0.68 ± 0.52
9.12 ± 3.04
3.13 ± 3.49
0.93 ± 0.86
0.57 ± 0.63
0.36 ± 0.46
-0.22 ± 0.95
0.76 ± 3.11
2.42 ± 9.12
0.4978
0.2712
0.2069
0.4058
0.2071
0.3071
0.3716
0.4163
0.1427
0.3336
0.3923
0.2910
0.3696
0.2435
0.4331
0.3953
0.1958
0.3275
3.81 ± 1.79
3.09 ± 1.24
0.73 ± 0.67
-0.93 ± 1.73
9.49 ± 5.27
6.31 ± 18.11
2.91 ± 1.22
2.55 ± 0.90
0.36 ± 0.47
-0.65 ± 0.65
9.04 ± 3.66
3.42 ± 5.69
0.90 ± 1.08
0.53 ± 0.66
0.37 ± 0.48
-0.25 ± 1.27
0.48 ± 2.95
2.91 ± 13.39
R5, resistance at 5 Hz; R20, resistance at 20 Hz; X5, reactance at 5 Hz; Fres, frequency of resonance; ALX, low-frequency reactance area.

## Slide 5
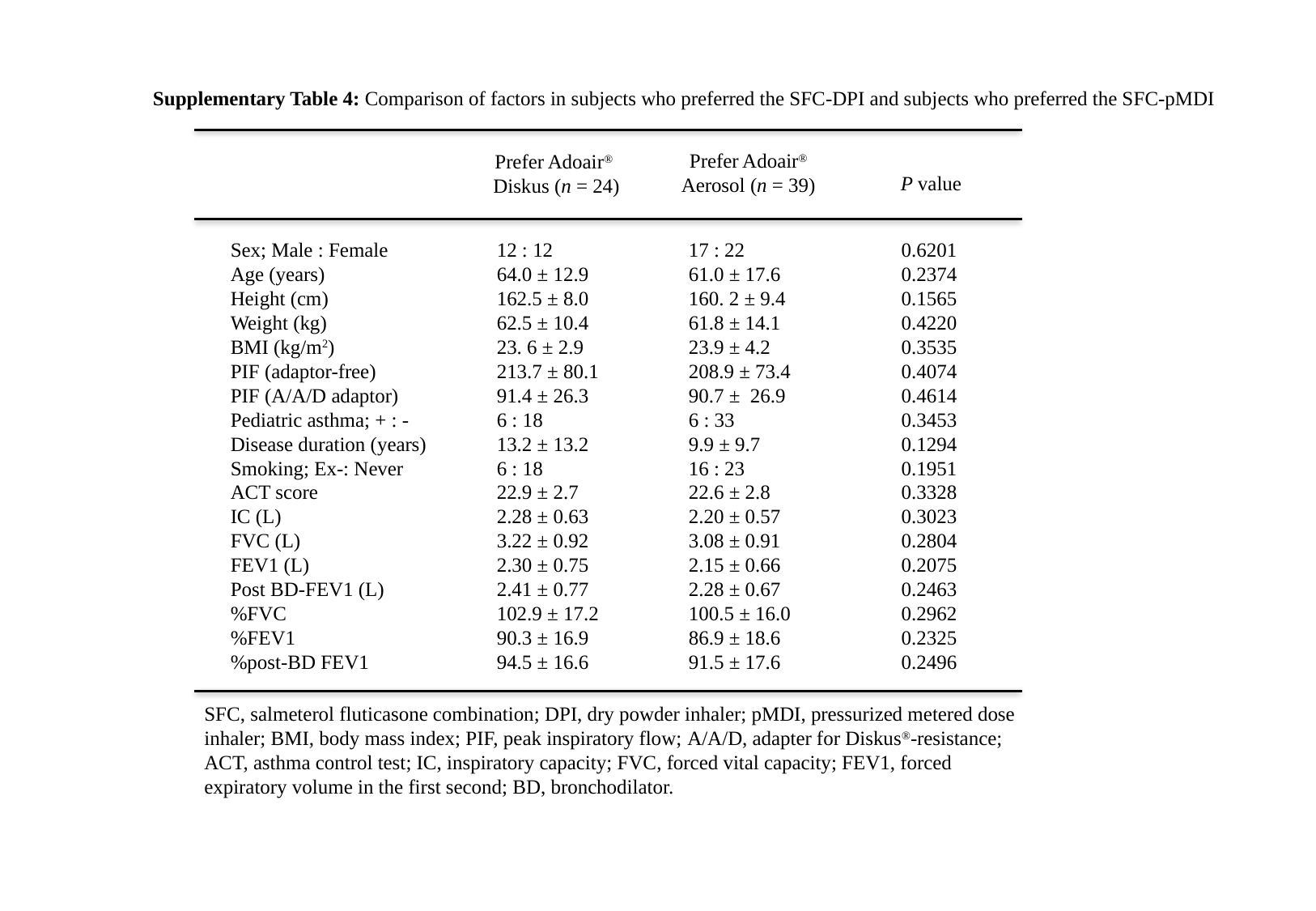

Supplementary Table 4: Comparison of factors in subjects who preferred the SFC-DPI and subjects who preferred the SFC-pMDI
Prefer Adoair®
Aerosol (n = 39)
Prefer Adoair®
Diskus (n = 24)
P value
Sex; Male : Female
Age (years)
Height (cm)
Weight (kg)
BMI (kg/m2)
PIF (adaptor-free)
PIF (A/A/D adaptor)
Pediatric asthma; + : -
Disease duration (years)
Smoking; Ex-: Never
ACT score
IC (L)
FVC (L)
FEV1 (L)
Post BD-FEV1 (L)
%FVC
%FEV1
%post-BD FEV1
12 : 12
64.0 ± 12.9
162.5 ± 8.0
62.5 ± 10.4
23. 6 ± 2.9
213.7 ± 80.1
91.4 ± 26.3
6 : 18
13.2 ± 13.2
6 : 18
22.9 ± 2.7
2.28 ± 0.63
3.22 ± 0.92
2.30 ± 0.75
2.41 ± 0.77
102.9 ± 17.2
90.3 ± 16.9
94.5 ± 16.6
17 : 22
61.0 ± 17.6
160. 2 ± 9.4
61.8 ± 14.1
23.9 ± 4.2
208.9 ± 73.4
90.7 ± 26.9
6 : 33
9.9 ± 9.7
16 : 23
22.6 ± 2.8
2.20 ± 0.57
3.08 ± 0.91
2.15 ± 0.66
2.28 ± 0.67
100.5 ± 16.0
86.9 ± 18.6
91.5 ± 17.6
0.6201
0.2374
0.1565
0.4220
0.3535
0.4074
0.4614
0.3453
0.1294
0.1951
0.3328
0.3023
0.2804
0.2075
0.2463
0.2962
0.2325
0.2496
SFC, salmeterol fluticasone combination; DPI, dry powder inhaler; pMDI, pressurized metered dose inhaler; BMI, body mass index; PIF, peak inspiratory flow; A/A/D, adapter for Diskus®-resistance; ACT, asthma control test; IC, inspiratory capacity; FVC, forced vital capacity; FEV1, forced expiratory volume in the first second; BD, bronchodilator.

## Slide 6
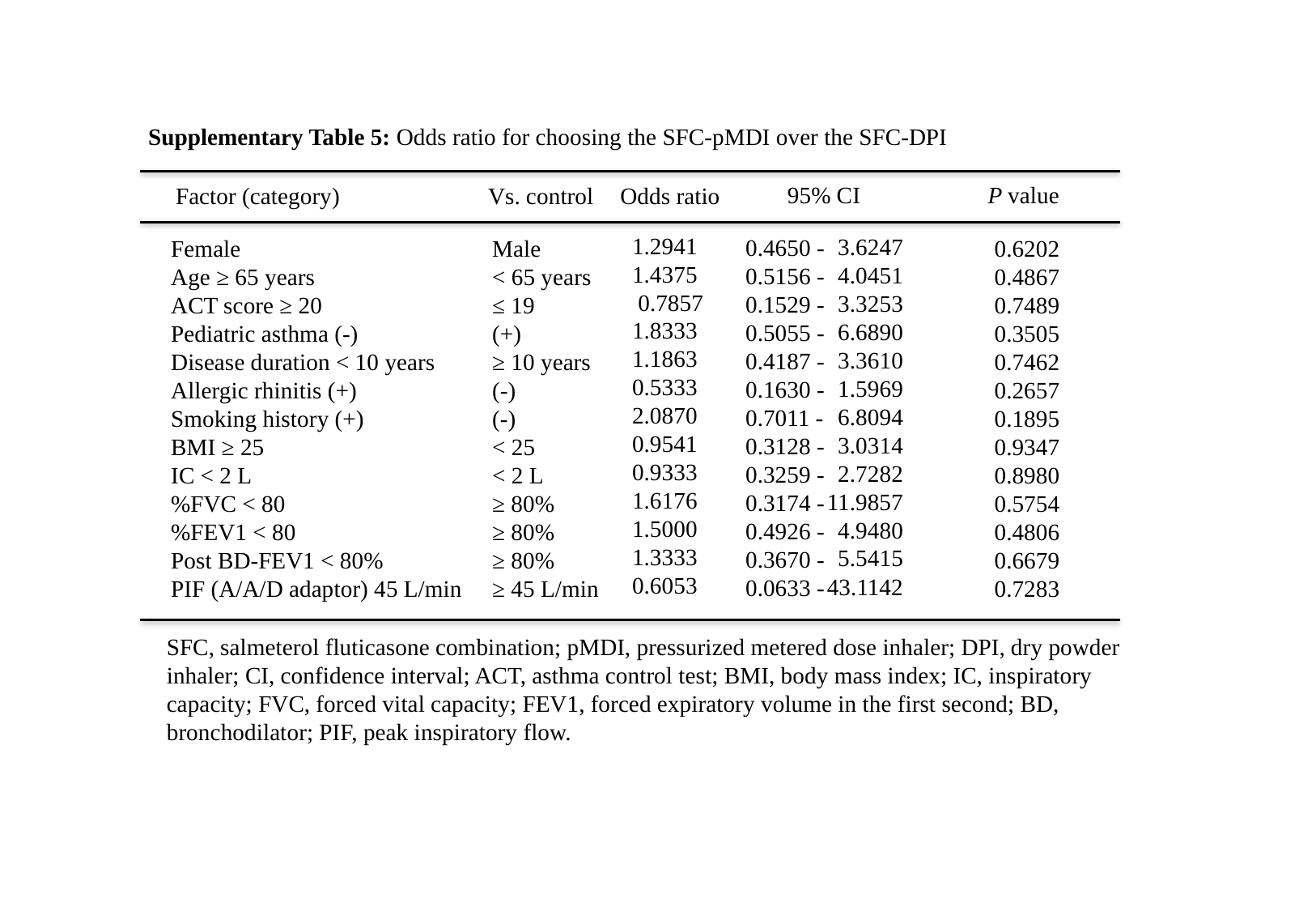

Supplementary Table 5: Odds ratio for choosing the SFC-pMDI over the SFC-DPI
95% CI
P value
Factor (category)
Vs. control
Odds ratio
1.2941
1.4375
 0.7857
1.8333
1.1863
0.5333
2.0870
0.9541
0.9333
1.6176
1.5000
1.3333
0.6053
3.6247
4.0451
3.3253
6.6890
3.3610
1.5969
6.8094
3.0314
2.7282
11.9857
4.9480
5.5415
43.1142
0.4650 -
0.5156 -
0.1529 -
0.5055 -
0.4187 -
0.1630 -
0.7011 -
0.3128 -
0.3259 -
0.3174 -
0.4926 -
0.3670 -
0.0633 -
Female
Age ≥ 65 years
ACT score ≥ 20
Pediatric asthma (-)
Disease duration < 10 years
Allergic rhinitis (+)
Smoking history (+)
BMI ≥ 25
IC < 2 L
%FVC < 80
%FEV1 < 80
Post BD-FEV1 < 80%
PIF (A/A/D adaptor) 45 L/min
0.6202
0.4867
0.7489
0.3505
0.7462
0.2657
0.1895
0.9347
0.8980
0.5754
0.4806
0.6679
0.7283
Male
< 65 years
≤ 19
(+)
≥ 10 years
(-)
(-)
< 25
< 2 L
≥ 80%
≥ 80%
≥ 80%
≥ 45 L/min
SFC, salmeterol fluticasone combination; pMDI, pressurized metered dose inhaler; DPI, dry powder inhaler; CI, confidence interval; ACT, asthma control test; BMI, body mass index; IC, inspiratory capacity; FVC, forced vital capacity; FEV1, forced expiratory volume in the first second; BD, bronchodilator; PIF, peak inspiratory flow.
